# The Role of Insulin Receptor Substrate Proteins in Bronchopulmonary Dysplasia and Asthma: New Potential Perspectives

**DOI:** 10.3390/ijms231710113

**Published:** 2022-09-04

**Authors:** Gokhan Gorgisen, Malik Aydin, Olivier Mboma, Mira Y. Gökyildirim, Cho-Ming Chao

**Affiliations:** 1 Department of Medical Genetics, Faculty of Medicine, Van Yüzüncü Yil University, Van 65080, Turkey; 2 Laboratory of Experimental Pediatric Pneumology and Allergology, Center for Biomedical Education and Research, School of Life Sciences (ZBAF), Faculty of Health, Witten/Herdecke University, 58455 Witten, Germany; 3 Center for Child and Adolescent Medicine, Center for Clinical and Translational Research (CCTR), Helios University Hospital Wuppertal, Witten/Herdecke University, 42283 Wuppertal, Germany; 4 Department of Pediatrics, University Medical Center Rostock, University of Rostock, 18057 Rostock, Germany; 5 Cardio-Pulmonary Institute (CPI), Universities of Giessen and Marburg Lung Center (UGMLC), Member of the German Center for Lung Research (DZL), Justus Liebig University Giessen, 35390 Giessen, Germany

**Keywords:** insulin receptor substrates, asthma, bronchopulmonary dysplasia, pediatric lung disease

## Abstract

Insulin receptor substrates (IRSs) are proteins that are involved in signaling through the insulin receptor (IR) and insulin-like growth factor (IGFR). They can also interact with other receptors including growth factor receptors. Thus, they represent a critical node for the transduction and regulation of multiple signaling pathways in response to extracellular stimuli. In addition, IRSs play a central role in processes such as inflammation, growth, metabolism, and proliferation. Previous studies have highlighted the role of IRS proteins in lung diseases, in particular asthma. Further, the members of the IRS family are the common proteins of the insulin growth factor signaling cascade involved in lung development and disrupted in bronchopulmonary dysplasia (BPD). However, there is no study focusing on the relationship between IRS proteins and BPD yet. Unfortunately, there is still a significant gap in knowledge in this field. Thus, in this review, we aimed to summarize the current knowledge with the major goal of exploring the possible roles of IRS in BPD and asthma to foster new perspectives for further investigations.

## 1. Introduction

Insulin receptor (IR) and insulin-like growth factor (IGF) receptor (IGFR) signaling regulate a variety of cellular processes including glucose metabolism, differentiation, and cell growth [1]. Although insulin and IGF are commonly involved in induction processes and overlapping signaling pathways due to shared downstream elements during biological processes, they may also harbor unique effects in cellular physiology [2]. Insulin and IGF signaling pathways are mainly known as metabolic regulators in the cell, but they are also implicated in allergic lung and developmental disorders related to metabolic diseases [3,4]. Further, recent studies have elaborated that the dysregulation of IGF signaling pathways may contribute to the disrupted development of the lung and lung disorders including lung cancer [5]. Common adaptor molecules that transmit extracellular signaling through IGFR and IR are insulin receptor substrate (IRS) proteins [6]. Although they were discovered to be substrates of IR, they can also interact with other receptors such as those for growth hormones, cytokines, integrins, and vascular endothelial growth factors (VEGF) [7]. They generate a critical node for the regulation of multiple signaling pathways depending on extracellular stimuli and signal diversion. In addition, they have pivotal roles in processes including inflammation, growth, metabolism, and proliferation [8]. Previous studies differentially emphasized the role of IRS proteins in the development of several diseases, e.g., allergic lung inflammation and diabetes [3,9,10,11]. However, there is still a significant gap in knowledge on how IRSs are related to the development of pediatric lung diseases such as asthma and bronchopulmonary dysplasia (BPD). This review summarizes the current evidence of IRS possibly being involved in asthma and BPD.

## 2. The Biological Role of Insulin Receptor Substrate Proteins in Human Tissue

The insulin receptor substrate family consists of six members, IRS1–6 [12]. All family members have evolutionary conserved two domains in their N-terminus. The Plecstrin homology (PH) domain modulates the interaction of IRS proteins with the cell membrane and induces the translocation of IRS1 from the cytosol to the nucleus through the nuclear localization signal located in the PH domain [13,14,15]. The Phosphotyrosine binding (PTB) domain is another conserved region located in the N-terminal of the IRS protein that plays a central role in the activation of IRS proteins after binding to tyrosine phosphorylated insulin and other receptors [13,14,15]. Moreover, there are insufficiently conserved regions at the carboxy-terminal ends of the IRS proteins. These contain various tyrosine, serine, and threonine motifs that regulate signal transduction and diversion [12]. This region contributes to the unique specificity owing to the unique regulation of each IRS protein. Importantly, IRS5 and IRS6 have a truncated C-terminal compared to the other members of the family [16,17,18].

In detail, the IRS members IRS1, IRS2, IRS5, and IRS6, are widely expressed in human tissues, whereas IRS4 presents a tissue-restricted pattern including the brain, thymus, and embryonic tissues. IRS3 acts as a pseudogene and is not expressed in humans [17,18,19,20,21].

**Figure 1:**
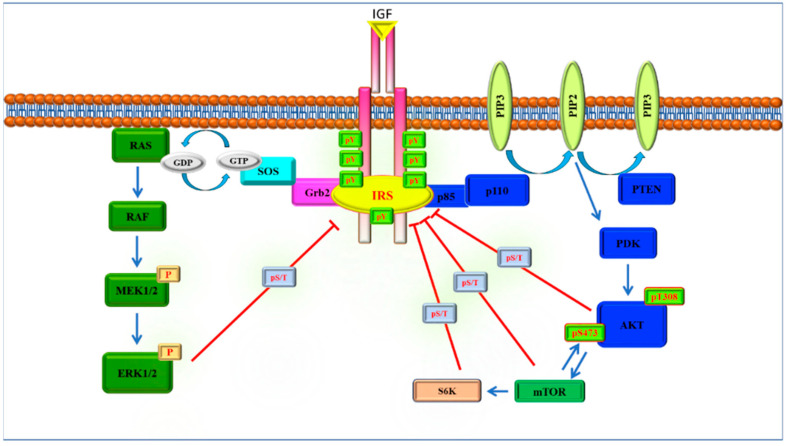
Canonical IRS signaling pathway adapted from [24]. IRS proteins mainly induce the activations of the PI3K-AKT and MAPK pathways. After the binding of receptor tyrosine kinases, IRS proteins are phosphorylated by their tyrosine residues and this activation triggers the IRS-induced signaling pathways. Activations of IRS proteins are regulated through Ser/Thr phosphorylations as a feedback mechanism of their downstream targets.

The IRS signaling pathway starts with the binding of ligands such as insulin and the insulin-like growth factor (IGF) to their corresponding receptors. This leads to autophosphorylation of the receptor via tyrosine residues (Figure 1) [22]. IRS proteins do not have kinase or other intrinsic enzymatic activity; however, they become phosphorylated by receptor tyrosine kinases following their interactions [23]. The tyrosine phosphorylation of IRS proteins triggers the transmission of signals from receptors to downstream targets in the cytosol. However, the tyrosine phosphorylation of IRS proteins mainly induces two signaling pathways including MAPK and PI3K-AKT [24]. 

In detail, the PI3K-AKT pathway is a critical node for cell signaling pathways [25]. It regulates many cellular processes such as metabolism, survival, and apoptosis [26]. IRS proteins contain YXXM motifs and phosphorylation of the tyrosine residues of the motifs YXXM leads to the activation of PI3K and the induction of mTOR and AKT activations (Figure 1) [24].

In addition to the PI3K-AKT pathway, upon activation of the IRS proteins, Grb2 binds to the YVNI motifs of the IRS proteins and triggers the activation of RAS-RAF-ERK1/2 signaling cascades (Figure 1) that regulate the expression of the genes responsible for cell proliferation and differentiation [27].

IRS proteins contain more than 50 S/T phosphorylation sites for the several IRS kinases at their COOH terminals [28,29]. In contrast to tyrosine phosphorylations, S/T phosphorylations usually inhibit signal transduction [10]. Under normal conditions, these phosphorylations balance each other, are well-regulated, and occur as a physiological negative feedback mechanism mainly by downstream targets of IRS proteins [29]. In addition to these downstream elements, other signaling mediators can also phosphorylate IRS proteins at S/T motifs such as TNF-α, NF-κB, and JNK [30,31,32,33]. The dysregulation of IRS S/T phosphorylations leads to the development of pathological conditions such as insulin resistance, Type 2-diabetes, cancer, and inflammatory diseases [11,24,33].

## 3. Possible Roles of IRS in Pediatric Lung Diseases

### 3.1. Pediatric Asthma

As a chronic inflammatory disease, asthma plays a special role among lung disorders. The exact etiology of asthma remains not fully understood, but it has a multifactorial and heterogeneous background where genetic and environmental factors have pivotal roles [34]. In recent decades, asthma has been increasingly recognized as a ‘syndrome’ with different etiologies and underlying pathophysiologic mechanisms, and it is subclassified into several subgroups or phenotypes [35,36,37,38,39,40]. Currently, one of the widest and best characterized endotype is allergic (eosinophilic) asthma with a type 2 immune response/inflammation, including type 2 T-helper cell lymphocytes (Th2) and other involved cell subsets [35,41]. However, chronic lower airway inflammation in asthma is caused by the infiltration of inflammatory cells, such as eosinophils, neutrophils, and T-helper cells, as well as the activation of mast cells. In addition, IgE production triggered by B lymphocytes and epithelial cell damage are some aspects occurring during the pathogenesis of asthma [41,42,43].

In detail, during the sensitization phase, antigen-presenting cells (APCs), mainly dendritic cells (DCs), internalize allergens that have passed the epithelial barrier and migrate toward the draining lymph nodes [41,44,45,46]. DCs present allergen-derived peptides to naive T-helper lymphocytes (CD4^+^) through major histocompatibility complex type II (MHC-II) in conjunction with costimulatory molecules such as CD80, CD86, and OX40L. Together with IL-4, this process triggers the differentiation of CD4^+^ to Th2 and T follicular helper cells (Tfh), which favors the humoral antibody production of IgE by inducing B cell isotypic commutation and plasma cell differentiation [41,45,46,47,48]. Th2 produces pro-inflammatory cytokines, also known as type 2 cytokines, which promote IgE production, activation of eosinophils (IL-5), mast cell development (IL-9), and airway hyperresponsiveness (IL-13) [41,46,47].

In the late phase, locally produced chemokines may cause the recruitment of macrophages, eosinophils, neutrophils, and Th2 lymphocytes [41,42,44]. The latter release specific cytokines such as IL-4, IL-5, IL-9, and IL-13, as well as a granulocyte-macrophage colony-stimulating factor (GM-CSF), which contribute to the maintenance of inflammation and can lead to remodeling, fibrosis, and hyperplasia [41,42,43,44,49,50,51].

Understanding the precise contribution of these signaling pathways to M2 macrophage polarization is a key factor in the search for potential therapeutic strategies to disrupt the proinflammatory signaling pathways in severe asthma and allergic diseases in the future [11,52,53].

### 3.2. Pediatric Asthma and IRS Signaling: A Forgotten Gap?

**Figure 2:**
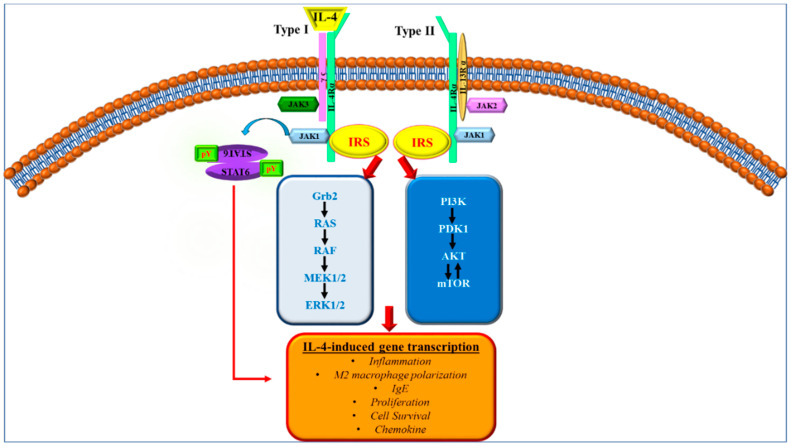
Interleukin-4-induced gene transcriptional effects through IRS and STAT6 activations. Upon binding of IL-4, tyrosine phosphorylated IL receptors generate binding sites for JAK1/2 and IRS proteins. JAK1/2 are mainly responsible for the activations of IRSs and STAT6 that trigger the IL4 -induced cellular effects such as inflammation, cell survival, and proliferation (adapted from [61]).

IL-4 and IL-13 have multiple important functions including the regulation of allergic responses [54]. As stated above, IRSs have been shown to be involved in allergic inflammation as well. Therefore, it is important to understand the interactions between IRSs, IL-4, and IL-13 proteins to explore the possible roles implicated in asthma. IRS1 was identified as a tyrosine-phosphorylated large molecular weight protein after insulin stimulation while IRS2 was determined in Factor Dependent Continuous-Paterson 2 (FDC-P2) cells after IL-4 treatment [55,56,57]. In IL-4 and IL-13 signaling, Janus kinase (JAK) proteins are required for the tyrosine phosphorylation of the IRS proteins (Figure 2) [58].

Recently, IRS2 has received increasing attention due to its role in the signaling cascade of type 2 cytokines, such as IL-4 and IL-13 [11,52]. IRS2, similar to its homolog IRS1, is an approximately 170 kDa adaptor protein that mediates the downstream signaling of receptor tyrosine kinases [11,59,60]. It is a member of the insulin receptor substrate family and is involved in signaling through IGF-1, insulin, and EPO, among others, and therefore plays a role in insulin-induced responses [11,59,60]. While its role in promoting insulin resistance and type II diabetes has been explored and its function as a regulator of M2 macrophage polarization has been described, not much is known about its precise contribution to the pathophysiology of asthma/allergy through the IL-4 pathway [11,52,60,61,62].

During IL-4 and IL-13 signal transductions, IL-4 binds to IL4Rα and induces the dimerization with the IL-4 γchain or IL13Rα1, while IL-13 interacts with IL13Rα1 and leads to the ‘heterodimerization’ of IL4R-α [63]. These complexes trigger the activation of JAK that phosphorylate the specific tyrosine motifs [63]. During these auto- and cross-phosphorylations, JAK1, JAK2, and JAK3 consequently interact with IL4R-α, IL13R-α1, and -γ-chain, respectively, which are crucial to present docking sites for IRS proteins [64]. The IRS proteins specifically bind to the tyrosine phosphorylated NPXY motif of the cytoplasmic tail of the receptors, which is also defined as the insulin and IL-4 receptor (I4R) motif [65]. The mutation of this motif may block the association between receptor and IRS proteins and inhibits the proliferation effect of IL4R-α signaling [65]. In the IL4R-α signaling pathway, IRS1 and IRS2 are activated by an IL-4 treatment [66]. The activation of IRS1 and IRS2 can trigger distinct signaling cascades including PI3K, AKT, and MAPK, which was already mentioned above. In addition to IRS proteins, STAT6 also binds to the IL4R-α chain upon ligand activation [67]. The activation of STAT6 induces the translocation of STAT6 from the cytosol to the nucleus and mainly regulates gene expressions [68] (Figure 2).

One of the downstream effectors of IRS2 is the p85α subunit of phosphatidylinositol 3-kinase (PI3K), which in turn activates several downstream signaling pathways and regulates epithelial cell migration, suggesting that IL-4 may have a beneficial effect on airway epithelial cell repair via recruitment of the IRS2 pathway [66]. Interestingly, further M2 gene expression, occurs only when activation of the IRS2 signaling pathways occurs via the type 1 IL-4 receptor rather than type II [53,62].

While tyrosine phosphorylation appears to activate IRS2 signaling, serine phosphorylation does the opposite by inhibiting p85α binding and PI3K activation and promoting IRS2 degradation [59,62]. The precise pathway involves two targets of rapamycin kinase complex 1 (TORC1)-activated proteins, namely GRB10 and p70S6K, the common γ-chain (γC), and IL-4Rα, and the latter initiating the serine phosphorylation of IRS2 [62]. The end result of these two negative regulatory mechanisms is a reduction in the M2 polarization of human macrophages in diseases such as asthma [62]. Another potential process for the downregulation of IRS2 is the Suppressors of Cytokine Signaling Protein Family (SOCS) 1 [53]. This protein family is induced by JAK-STAT activation, nuclear displacement of Signal Transducers and Activators of Transcription (STAT)6, and intracellular signaling through PI3K [53]. SOCS1 inhibits a negative feedback loop by forming an E3 ubiquitin ligase with other proteins, increasing the poly-ubiquitination of phosphorylated IRS2 and promoting the proteasomal degradation of IRS2 [53]. In addition, SOCS1 expression in response to IL-4 appears to be decreased in allergic individuals compared with healthy individuals [53]. 

### 3.3. Bronchopulmonary Dysplasia

Bronchopulmonary dysplasia is the most common pulmonary complication in infants born before 30 weeks of gestational age and contributes to long-term morbidity and mortality [69]. According to the severity-based definition of BPD, up to 30–40% of preterm neonates born with a gestational age ≤ 28 weeks suffer from BPD [69]. The pathogenesis of this chronic pulmonary disease is multifactorial and most commonly seen in premature infants undergoing mechanical ventilation with oxygen therapy (old BPD). Furthermore, other risk factors such as intrauterine growth restrictions and pre-/postnatal infections may lead to a pulmonary growth arrest resulting in alveolar simplification and pulmonary vasculature disturbance [70,71,72]. Clinically, BPD is defined by the need for supplemental oxygen or ventilator support at day 28 of life or 36 weeks of gestational age [73]. Despite numerous advances in neonatal care leading to improvement in the survival of preterm infants, options for the prevention and treatment of BPD are still very limited, and curative therapy is still lacking [74]. The development of exogenous surfactant protein application supporting preterm infants to breathe postnatally allowed a less injurious ventilation strategy and has changed the histomorphological phenotype of BPD (new BPD) defined by a decrease in alveologenesis and reduction in small vessel development, alterations in the growth factor signaling, and extracellular matrix changes [72,75].

**Figure 3:**
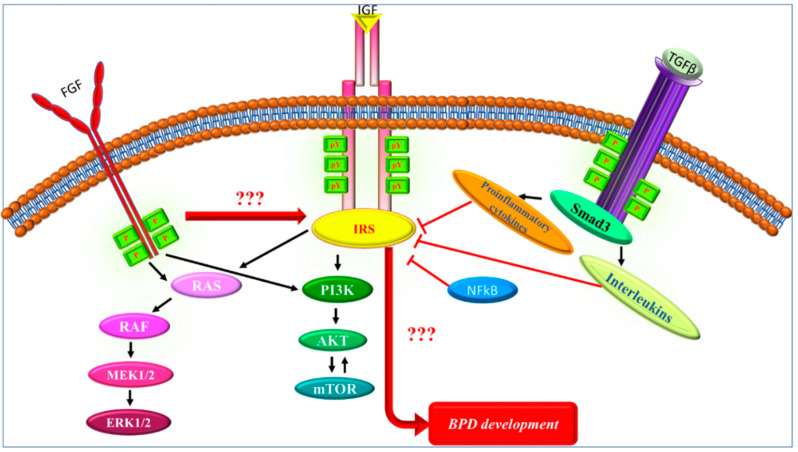
Hypothetical role of IRS proteins in BPD development. IGFR, FGF, and TGF-β signaling pathways have pivotal roles in BPD. IRS proteins are the common proteins of these signaling pathways. In addition to the downstream targets of IRS proteins, IRS-related signaling pathway members such as proinflammatory cytokines, NF-κB, and interleukins inhibit IRS signaling through Ser/Thr phosphorylations. These inhibitions may have an important role in the development of BPD.

Hyperoxic injury is one of the main risk factors in BPD pathogenesis, which is thought to disrupt critical signaling pathways that induce lung development, including branching and septation [76]. Several signaling pathways contributing to these processes have been described, such as IGFR [5], FGF10 [77,78], and TGF-β [79] signaling pathways, where the IRS proteins might serve as a common component (Figure 3) [80,81].

### 3.4. Aspects of IRS Signaling Possibly Involved in BPD

IRS proteins are the main adaptor molecules of IGF signaling [8,82]. One of the main risk factors of BPD is hyperoxia. Hyperoxia interferes with IGF-1 signaling [5,83,84] and inhibits IGF-1/IGF-1R signal transduction by decreasing the binding affinity in the lungs of preterm infants or the distal epithelial cells in fetal lungs [5]. Decreasing the binding affinity of IGF results in impaired proliferation, formation of secondary ridges, and alveologenesis [85,86]. Although it may seem paradoxical at first, in vivo experiments have shown that mice are protected from 90% oxygen-induced lung injury when IGF-1R expression is reduced and leads to less edema, vascular extravasation, and respiratory failure compared to control mice [87].

IGF signaling is important for normal lung development. Thus, in in vivo experiments, components of IGF signaling were shown to have different expression patterns in different compartments during embryogenesis [88].

Serum IGF-1 levels are significantly reduced in patients with BPD [89]. However, the expression of IGF-1 in epithelial mucosal fluid, epithelial cells, and peribronchial myofibroblasts was increased in BPD [90,91]. This was also demonstrated in the hyperoxia-stimulated ex vivo model of neonatal rat lung. The IGF-1 staining was most pronounced in the airway and alveolar epithelial cells [85]. The cell proliferation was associated with increased IGF-1 mRNA and protein expression and could be inhibited by IGF-1 antibody [85].

It has been shown in previous studies that the downstream targets of IGF signaling play a role in the development of BPD [92]. However, the effect of IRS in relation to IGF in the context of BPD has not been explored. The PI3K-AKT pathway is one downstream target of IGF signaling (Figure 3) [5]. Several studies have shown the relevance of PI3K in parenchymal cells in lung disease [93]. In particular, in adult respiratory distress syndrome (ARDS), the PI3K-dependent activation of PKB in lung endothelial cells was shown to be triggered by overventilation [93]. This overventilation causes a PI3K-dependent shift of NF-κB which can decrease *Fgf10* expression during the development of BPD [94,95,96].

It is also known that the activation of PI3K/AKT via hydrogen has a protective effect on alveolar epithelial cells type 2 (AEC2) during hyperoxia treatment in animal experiments [97].

The mammalian target of rapamycin (mTOR) is a part of the PI3K/AKT pathway and can be induced by hyperoxia [98,99,100,101]. It was shown that inhibition of the mTOR signaling pathway suppresses the proliferation of lung fibroblasts [102]. It is also described that blocking from the mTOR pathway could downregulate the TGF-β expression [103]. 

ERK1/2 is the other main downstream target of IGF signaling (Figure 3). It has been demonstrated that hyperoxia can activate ERK1/2 in in vivo models [104,105].

*Fgf10* is one of the most significant developmental genes expressed in the submesothelial mesenchyme of the developing lung [106,107]. *Fgf10* encodes a secreted diffusible protein that acts in a paracrine manner through the epithelial receptor Fgfr2b [108,109]. Most important, FGF10 has been shown to be downregulated in children suffering from fatal severe BPD [110]. There is growing evidence supporting a close interaction between lung vasculature and branching morphogenesis via endothelial-epithelial crosstalk [77,111,112]. It was also demonstrated that Fgf10 is critical for the differentiation of the Fgf10-positive progenitor cells towards the lipofibroblast lineage, a subset of fibroblast believed to play an important role in de novo alveologenesis during regeneration after lung injury [113]. Although previous studies have shown that FGF family members induce the activation of IRS1 and IRS2 proteins through FGFR and IGFR receptors, we do not yet know the exact role of IRS proteins in the development of BPD through the activation of these receptors [114]. Other studies mainly focused on the role of FGF and IRS1 signaling pathways in cancer and different diseases [115,116]. In MCF7 cells, after treatment with FGF, IRS1 expression showed a rapidly increasing profile [117]. In a study regarding the role of insulin resistance in chronic liver disease, Manzano-Nunez and colleagues revealed IRS2 as a positive regulator for the intercellular interaction and the transition from stromal to epithelial repair via Fgf7-Fgfr2b signaling [118]. 

TGF-β takes a central role in postnatal lung development and alveologenesis. TGF-β signaling is activated by the binding of TGF-β to the type II TGF-β receptor (TβRII) [119]. This complex subsequently binds to one of two variants of the type I receptor (ALK-1 or ALK-5) [119]. The type I receptor transmits signals into the cell by the second-messenger SMAD proteins, namely SMAD1 m and SMAD3 [119,120]. This happens in combination with the co-SMAD, SMAD4, or by SMAD-independent pathway [119,120]. In vivo experiments have revealed that TGF-β also acts as a positive regulator of branching and alveologenesis [121,122,123]. However, experiments in various in vivo models have shown that the increased expression of TGF-β and activation of the TGF-β signaling pathways are associated with the onset and development of BPD [124,125,126,127].

As increased pulmonary TGF-β expression precedes most BPD-related pathophysiological manifestations and infiltration of neutrophils and monocytes into the lungs occurs during disease development [127,128,129]. These recruited monocytes and macrophages are the main source of TGF-β [130], which leads to an increase in secreted pro-inflammatory cytokines such as many interleukins (IL), e.g., IL-1β, IL-6, IL-8, and TNF-α [79,131,132]. The development of pulmonary edema is also associated with anti-inflammatory factors, including IL-4, IL-10, IL-12, and IL-13 or the IL-1 receptor antagonist [133,134,135]. A previous study showed that IRS1 and IRS2 are the key molecules in the TβR-V/LRP-1-induced growth inhibition in mink lung epithelial cells [136]. Another study that focused on the interaction between TGF-β and IRS signaling showed that IRS1 may suppress TGF-β-induced epithelial-mesenchymal transition in A549 cells (non-small cell lung cancer cell line) through inhibition of Snail and Slug expressions [137]. In contrast, TGF-β inhibits cell proliferation and increases apoptosis via inhibition of IRS1 expression and activation in colon cancer cells [138]. All these studies focused on cancer development and mainly showed the negative regulation between IRS signaling and TGF-β signaling. This regulation may also have a role in the development of BPD. 

Although IRS family members are the common proteins for the IGF signaling cascade, there is no study that focuses on the relationship between IRS proteins and BPD. Previous studies showed that ERK1/2, AKT, and mTOR phosphorylate IRS proteins at Ser/Thr motifs and negatively regulate the IRS protein’s actions (Figure 3). Therefore, IRS proteins may have a pivotal role in the development of BPD, but this needs to be further investigated.

## 4. Conclusions

It has been extensively shown that IRS proteins are major players involved in many processes such as inflammation, growth, metabolism, and proliferation. However, their roles in lung disorders are insufficiently understood. Here, we have comprehensively summarized the current knowledge on how IRS might be possibly involved in the pathogenesis of asthma and BPD, focusing on the signaling pathways of IGF, IL-4, FGF, and TGF- β receptors. Even though some of the evidence is still weak and hypothetical, it allows new perspectives for further investigations in order to shed more light on the role of IRS in asthma, BPD, and other pediatric lung diseases.

## Data Availability

Not applicable.

## Abbreviations

AEC2 = alveolar epithelial cell type 2; APCs = antigen-presenting cells; BPD = bronchopulmonary dysplasia; DCs = dendritic cells; FDC-P2 = Factor Dependent Continuous-Paterson 2; GM-CSF = granulocyte-macrophage colony-stimulating factor; I4R = IL-4 receptor; IGFR = insulin-like growth factor receptor; IR = Insulin receptor; IRS= insulin receptor substrates; JAK = Janus kinase; mTOR = Mammalian target of rapamycin; PH = Plecstrin homology; PTB = Phosphotyrosine binding; SOCS = Suppressors of Cytokine Signaling Protein Family; STAT = Signal Transducers and Activators of Transcription; TβRII = TGF-β receptor; Tfh = T follicular helper cells; Th2 = type 2 T-helper cell lymphocyte; TORC1 = target of rapamycin kinase complex 1; TYK = tyrosine kinases; VEGF = Vascular endothelial growth factor
